# *Galega officinalis* extract regulate the diabetes mellitus related violations of proliferation, functions and apoptosis of leukocytes

**DOI:** 10.1186/s12906-017-2079-3

**Published:** 2018-01-08

**Authors:** Mariia Nagalievska, Mariya Sabadashka, Halyna Hachkova, Nataliia Sybirna

**Affiliations:** Department of Biochemistry, Faculty of Biology, Ivan Franko National University of Lviv, 4, Hrushevskyi St, Lviv, 79005 Ukraine

**Keywords:** *Galega officinalis* L., Diabetes mellitus, Immunocompetent cells

## Abstract

**Background:**

An impaired leukocytes function is the factor causing the susceptibility of patients with diabetes mellitus to infections. The outmost importance for the understanding of the immunological processes involved in diabetes pathogenesis is to give the characteritics of the immunological profile and changes therein, during the course of desease. Long-used in folk medicine to treat diabetes *Galega officinalis* L. has been chosen for the correction of the immune system dysfunction.

**Methods:**

The experiments were conducted on male Wistar rats. Fractionation of bone marrow cells suspension was performed in a three-layer ficoll–sodium amidotrizoate density gradient. The lymphocytic-granulocytic cells proliferative activity was studied using enzyme immunoassay with 5-bromo-2′-deoxyuridine (BrdU). For staining of bone marrow preparations May-Gruenwald-Romanowsky-Giemsa (Pappenheim) method was used. To evaluate the content of cationic proteins and myeloperoxidase in neutrophilic leukocytes cytochemical studies were performed. Content of tumor necrosis factor alpha was carried out by immuno-enzymatic analysis. Lymphocytes apoptosis was examined by fluorescent analysis using annexin V.

**Results:**

Diabetes mellitus development was accompanied with violation of neutrophils and lymphocytes proliferation, increased activity of myeloperoxidase and enhanced apoptosis process. Administration of *Galega officinalis* extract under the condition of diabetes promotes the restoration of neutrophils bone marrow pool and the reduction of lymphoblasts number and causes inhibition of the lymphocytes apoptosis process.

**Conclusions:**

Investigated medicine has a pronounced immunocorrective effect under the conditions of diabetes mellitus and can become the basis for creating a new generation of antidiabetic drugs.

## Background

The past decade has accumulated the evidence suggesting that the immune system plays an important role in the pathogenesis of insulin-dependent diabetes mellitus (DM) [1]. A number of patients with DM have increased in incidence of infections. Some of these infections are also more likely to have a complicated course in diabetic than in nondiabetic patients [2]. Diabetes related complications are reflected in the disruption of host defense mechanisms, from non-specific to specific immune systems. The inhibition of the proliferative response to different stimuli in the lymphocytes of diabetics with poorly controlled disease was observed [3].

The quality and the magnitude of an immune response are determined by the communication between cells of immune system [4].

An inflammatory disorder such as diabetes is accompanied with implicated innate lymphocytes functions, leading to tissue dysfunction, barrier breach and severe pathology during local infection. Adaptive immune responses could be modulated by these cells by releasing of soluble meditators and cell contact-dependent interactions, or by effecting on antigen-presenting cells and stromal cells. Produced by innate lymphocytes T helper cell cytokines modulate the number and activity of macrophages, eosinophils, neutrophils, and dendritic cells and directly influencing T cells. During inflammatory disorders tissue-specific immune responses to repeated exposure of pathogens may be coordinated by T cells and innate lymphocytes interactions [5].

Impaired leukocytes functions are the factors causing the susceptibility to infections in patients with DM. Hence, the characteristics of the immunological profile and changes therein in the course of disease are of outmost importance for the understanding immunological processes involved in DM pathogenesis. In addition, the search of effective drugs ensuring the silence and/or modulation of the immune response, preferably without negative effects is the most promising area of study.

Therefore for the correction of the immune system dysfunction *Galega officinalis* L., which has long been used in folk medicine to treat diabetes, has been chosen. The preliminary research established that administration of *Galega officinalis* extract at dose 600 mg / kg per day reveals the marked hypoglycemic effect under the DM condition. It has shown the mobilization of antioxidant and antiradical protection mechanisms. At the same time this extract had a corrective influence on the leukocyte differential count. *Galega officinalis* extract revealed the inhibitory effect on the genetically programmed cell death that is evidenced by the normalization of number of white blood cells containing apoptosis regulatory proteins (p53 and Bcl-2), and poly-(ADP)-rybosylated proteins in rats leukocytes [6–8]. A lack of information on the biologically active substances isolated from *Galega officinalis* that posses immunomodulatory activity was noted in the analyzed literature data.

Therefore, the aim of this study is to provide qualitative and quantitative analysis of biologically active substances in chloroform fraction of *Galega officinalis* extract and research the effect of extract on the leukocytes differentiation (level of thymidine analogue 5-bromo-2′-deoxyuridine inclusion in proliferating bone marrow cells, leukocytes precursors population in bone marrow), neutrophils functional activity (myeloperoxidase and cationic proteins content) and lymphocytes apoptosis under the experimental diabetes mellitus type 1 that was induced by intraabdominal injection of streptozotocin.

## Methods

### Plant material

The aerial (leaves and stems) parts of *Galega officinalis* was collected from Lviv, Ukraine in June 2013. The taxonomic identification was made by the curator of medicinal plants collection of Botanical Garden of Ivan Franko Lviv National University, Senior Research Fellow Mariia Skybitska. A voucher specimen has been deposited at the Herbarium of the Department of Botany, Ivan Franko Lviv National University, Lviv, Ukraine.

### Preparation of *Galega officinalis* extract and compounds identification [9, 10]

*Galega officinalis*, introduced in the Botanical Garden of Ivan Franko Lviv National University, was used for studies. Aerial (leaves and stems) part was collected during flowering then it was dried out, homogenized and ethanol extract was produced by infusion in 96% ethanol for 12 h at a ratio of 1:5 at room temperature. Ethanol extract was evaporated in vacuum using rotary evaporators LABOROTA 4001 (Heidolph, Germany) at the temperature of 50–55 °C to obtain dense residue extract of jam consistency. Percentage yield of the crude extract was 15–17%. To evaporated original ethanol extract an equal volume of water and chloroform were added. After shaking samples were centrifuged for 10 min at 600 g. The obtained chloroform fraction was evaporated in vacuum at a temperature of 50–55 °C to obtain solid residue. Percentage yield of the chloroform fraction was 3.3–5%. Stabilization of chloroform fraction was performed by adding biocomplex PS (surface-active products of *Pseudomonas sp*. PS-17 biosynthesis at a concentration of 0.6 g / l), to the initial mixture, obtained by adding water to chloroform fraction of *Galega officinalis* extract, followed by shaking in Vortex (Biosan, Latvia). This method of extraction provides a stable water emulsion deprived of toxic alkaloids. For the research stabilized water emulsion of chloroform fraction, with which control animals and animals with DM were treated for 2 weeks orally daily at same time.

Chloroform fraction of extract was analyzed using an Agilent Technology 6890 N chromatograph with mass spectrometer detector 5979В. Conditions of analysis: chromatographic capillary column HP-5MS 30 m in length and inner diameter of 250 μm, 0.25 μm phase. Helium was used as carrier gas at a constant flow rate of 1.5 ml / min and sample volume of 1 ml. Injector 7683В, Split 20:1, evaporator temperature 250 °C. Thermostat programmed temperature of 75 °C (over 2 min) with heating 15 °C / min to 300 °C (within 9 min). Mass selective detector, interface temperature of 280 °C, ionization by electron impact, ionization energy 70 eV, ion source temperature of 230 °C, the quadrupoles temperature 150 °C. Total duration of gas chromatography was 24 min. The relative percentage of the amount of each component is calculated by comparing its average peak area to the total area. Identification was performed by comparing the mass spectra data with mass spectral libraries NIST05a and WILEY.

### Experimental animals

The experiments were conducted using three-month-old male Wistar rats weighing 150 to 220 g. The animals were maintained in clean and dry polypropylene cages in controlled temperature of 25 ± 2 °C and 45–55% relative humidity and a 12-h dark-light cycle in the animal house (Ivan Franko National University of Lviv). The rats were fed with a standard laboratory diet and water ad libitum. The rats were acclimatized at least 7 days to adapt to their environment before any experimental manipulation. Food was withdrawn 12 h prior to and during the experiment. General health status of the rats was monitored on alternate days, and no adverse events were recorded during the housing period. The protocol used in this study was carried out with the guidelines of the according to the “General ethical principles of experiments on animals”, adopted at the I National Congress on Bioethics (Kyiv, 2001) and the European Convention for the Protection of Vertebrate Animals Used for Experimental and Other Scientific Purposes (Strasbourg, France, 1986) and approval was taken from ethical committee of Ivan Franko National University of Lviv, Ukraine. Animals were randomly divided into following groups (*n* = 5–8/group): control animals (C); control animals that were treated with extract of *Galega officinalis* at dose 600 mg / kg per day (C + G); animals with diabetes mellitus (D); animals with diabetes that were treated with extract of *Galega officinalis* at dose 600 mg / kg per day (D + G). All the samples from animals subjected to the treatments were included in the data analysis. Animals from group C + G and D + G were receving stabilized water emulsion of chloroform fraction *Galega officinalis* extract through a tube, animals from group C and D were receving water at same way and period of a day.

### Induction of diabetes

Following an overnight starvation, diabetes was induced by intraabdominal injection of streptozotocin (Sigma, USA) dissolved before use in 10 mM citrate buffer (pH 5.5) at a dose of 0,055 g/kg body weight. The fasting blood glucose level was measured after 3 days to assess the development of DM. The studied medicines started to be administered on the 14 day after induction of diabetes.

### Biochemical analysis

The level of glucose in plasma was determined by glucoseoxidase method using a commercially available kit (Filisit diagnostics, Ukraine). The animals with glucose concentration 10 mmol / l and higher were used in conducted research. The level of glucose in plasma was determined as a routine procedure with the purpose of accurate testing of rats belonging to groups of diabetic animals. These results are not shown in the article.

### Collection of blood and blood leukocytes separation

At the end of the experimental period, the rats were starved for 15 h then anesthetized using deep diethyl ether anesthesia method and euthanized by decapitation. Whole blood was collected and immediately transferred to heparinized tubes. Leucocytes were separated on gradient of Histopaque-1083 (density of 1.083 g/ml) (Sigma, USA).

### Staining of bone marrow preparations using May-Gruenwald-Romanowsky-Giemsa (Pappenheim) method [11, 12]

May-Gruenwald dye solution was applied to dry bone marrow smear. After 3 min the equivalent amount of distilled water was added and thoroughly mixed. When smear became pink in color, dye was poured and freshly prepared Romanowsky-Giemsa dye was added to still wet bone marrow smear. After 8–15 min, dye was poured and smear was washed with water. All bone marrow cells (at least 500) were counted in several areas of the smear and the percentage of neutrophils and lymphocytes precursors were calculated. Total amount of lymphocytes precursors were adopted as 100%.

### Proliferative activity of bone marrow lymphocytic-granulocytic cells assay

The lymphocytic-granulocytic cells proliferative activity was studied using enzyme immunoassay. This assay allows making a quantitative assessment of the level of thymidine analogue – 5-bromo-2′-deoxyuridine (BrdU) inclusion in nucleic acids of proliferating cells. Animals were injected intraperitoneally with BrdU (Millipore, USA) at the dose of 50 g / kg body weight. 12 h after BrdU injection animals were decapitated and femurs were isolated for further aspiration of the bone marrow cell suspension. Fractionation of bone marrow cells suspension was performed in a three-layer ficoll–sodium amidotrizoate density gradient [12]. The density gradient was prepared by alternate layering of ficoll–sodium amidotrizoate solutions in the following order: ρ3 = 1,11 g / cm^3^, ρ2 = 1,09 g / cm^3^ and ρ1 = 1,03 g / cm^3^. The bone marrow cells suspension was applied to the top layer, after which the tubes were centrifuged for 10 min at 500 g. In this case, the cells concentrated at the boundaries of separating gradient solutions at different layers: lymphoid, erythroid and granulocyte-monocyte populations. Populations of lymphoid and granulocyte-monocyte cells were joined and then were washed with physiological saline buffer and were used for the further research. Proliferative activity was determined according to the protocol of the manufacturer set “Brdu Cell Proliferation Assay” (Millipore, USA).

### Determination of cationic proteins content in neutrophils from blood [13]

Nonenzyme cationic proteins are localized in a specific and an azurophilic granules mainly in neutrophilic leukocytes. Chemical properties of cationic protein are caused by the large number of positive charge NH_2_-groups in molecules. To determine cationic proteins content anionic dyes (bromphenol blue) were used. Cationic proteins were detected at cytoplasm in the form of blue pellets. The number of cells with blue pellets was counted microscopically.

### Determination of myeloperoxidase level in neutrophils from blood by Graham-Knol method [14]

Myeloperoxidase is a lysosomal enzyme that catalyzes the oxidation of various substrates in the presence of hydrogen peroxide. It is predominantly localized in specific azurophilic granules in the cytoplasm of granulocyte and is a common marker of myeloid cells. In the presence of myeloperoxidase benzidine is oxidizes by hydrogen peroxide with formation of brown oxybenzidine. Myeloperoxidase was detected in cytoplasm as a brown pellet. The number of cells with brown pellet was counted microscopically.

### Evaluation of cytochemical studies [15]

Evaluation of 2.11 and 2.12 cytochemical studies were performed by semiquantitative method using Astraldi principle that is based on the differentiation of a specific color varying intensity (0, +, ++, +++). The results were expressed as the average cytochemical coefficient (ACC) – that was calculated using the formula:

$$ ACC=\frac{\mathrm{A} \times 0+\mathrm{B} \times 1+\mathrm{C} \times 2+\mathrm{D}\times 3}{\mathrm{n}}, $$

where A – a number of cells with negative (0) reaction, B – a number of cells with poorly positive (+) reaction, C – a number of cells with moderately positive (++) reaction, D – a number of cells with sharply positive (+++) reaction, n – a number of counted cells. At least 200 cells were counted.

### Immuno-enzymatic analysis of tumor necrosis factor alpha contents in plasma

The content of TNF-α in rats plasma were determined by immuno-enzymatic analysis using standard ELISA set (Sigma, USA) according to the protocol of the manufacturer set.

### Fluorescent analysis of lymphocytes apoptosis by binding with Annexin V

Characteristic biochemical feature of apoptosis at cell plasma membrane level is translocation of phosphatidylserine (PS) residues from the inner to the outer side of membrane [16]. Phospholipid binding protein annexin V, conjugated with fluorochrome - fluorescein isothiocyanate (annexin V-FITC, FITC excitation wavelength - 494 nm and emission 518 nm), was used to identify the PS exposure on the cell surface. Whereas the integrity of the membrane is violated annexin binds to PS of internal lipid monolayer, for registration of cell membranes permeability supravital dye propidium iodide (PI) was used. PI, when penetrated to the cell, binds to the DNA molecule and forms a compound that fluoresces in the red region of the visible spectrum (excitation wavelength 536 nm and emission 617 nm). Thus, the double fluorescent staining of cells with annexin V-FITC and PI makes it possible to estimate the number of live cells and cells at early and late stages of apoptosis. In this work a set of reagents Annexin V-FITC Apoptosis Detection Kit, «BioVision» (USA) was used. After washing with PBS 2 × 10^5^ leukocytes were added to 0.1 ml of buffer (10 mM HEPES / NaOH, pH 7.4, 140 mM NaCl and 2.5 mM CaCl_2_) to which 5 μl Annexin V-FITC (working concentration of 2.5 μg / ml) and 5 μl PI (working concentration of 0.5 μg / ml) were added. Cells were incubated for 15 min in the dark at room temperature, after which 0.4 ml of buffer was added to the suspension. Cytometric analysis of lymphocyte was performed on flow cytometer FACSCalibur (Becton Dickinson, USA). In the leukocytes suspension sample the following parameters were evaluated: forward scattered light (FSC) (characterizing the size of cells), side scattered light (SSC) (characterizing optical heterogeneity of cytoplasm, nature of cellular inclusions and granularity of cells) and intensity of Annexin V-FITS (FL1) and PI (FL3) fluorescence. Lymphocytes population was selected by FSC vs. SSC gating. After the exclusion of debris (in FSC and SSC parameters) and the allocation of lymphocytic gate, the amount of Annexin^+^- and PI^+^-cells in DotPlot mode was determined. Discriminant analysis of the type of cell death included: 1st quadrant region - annexin V^−^/PI^+^ − cells with features of necrosis; 2nd quadrant region - annexin V^+^/PI^+^ − cells with features of late apoptosis; 3rd quadrant region - annexin V^−^/ PI^−^ - viable cells; 4th quadrant region - annexin V^+^/PI^−^ - cells with features of early apoptosis (Fig. 4). All statistical calculations and computations were performed with Cytomation Summit (MoFlo, USA).

### Statistical analysis of results

Statistical analysis of the results was carried out using Origin Pro. The calculation of basic statistical parameters was performed by direct quantitative data obtained from the study (arithmetic mean – M, the standard deviation of the arithmetic mean – m). To assess the reliability of the difference between statistical characteristics of the two alternative data sets, we performed one-way analysis of variance. The difference was considered significant under *p* ≥ 0.95 (the level of significance *P* < 0.05).

## Results

**Fig. 1:**
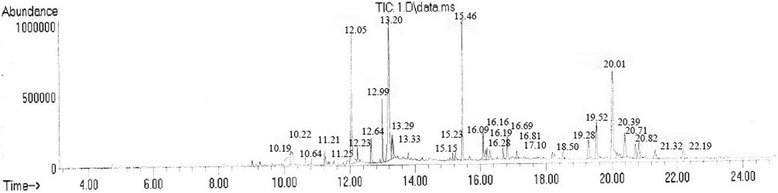
Chromatogram of biologically active substances in chloroform fraction of *Galega officinalis* extract

Gas chromatography/mass spectrometry method allowed to identify 31 components in *Galega officinalis* extract [17]. Most of the components has been identified and presented in Table 1 and Fig. 1.

**Table 1:** Qualitative and quantitative analysis of biologically active substances in chloroform fraction of *Galega officinalis* extract

Peak no	R.T., min.	Name of the compound	Peak Area %
1.	10,186	Not identified	5,24
2.	10,222	MOME inositol	2,08
3.	10,638	Tetradecanoic acid	0,74
4.	11,215	Neophytadiene	0,83
5.	11,251	2-pentadecanone, 6, 10, 14-trimethyl	0,58
6.	12.053	Hexadecanoic acid, ethyl ester	15,79
7.	12.232	Hexadecanoic acid, ethyl ester	0,85
8.	12.642	Not identified	2,06
9.	12.999	Phytol	3,62
10.	13.201	9,12,15-Octadecatrienoic acid, methyl ester, (Z,Z,Z)-	17,82
11.	13.290	Octadecanoic acid	1,67
12.	13.332	9,12,15-Octadecatrien-1-ol, (Z,Z,Z)-	1,52
13.	15.151	Not identified	0,66
14.	15.235	Hexadecanoic acid, 2-hydroxy-1-(hydroxymethyl)ethyl ester	0,95
15.	15.455	Phthalic acid, 2-ethylhexyl isohexyl ester	8,33
16.	16.085	2H-1-Benzopyran-7-ol, 3-(2,4-dimethoxyphenyl)-3,4-dihydro-	1,88
17.	16.162	Eicosane	0,83
18.	16.198	Nonanoic acid, 9-(3-hexenylidenecyclopropylidene)-,2-hydroxy-1-(hydroxymethyl)ethyl ester (Z,Z,Z)-	1,28
19.	16.287	6a,12a–dihydro-6H-(1,3)dioxolo(5,6)benzofuro(3,2-c)chromen-3-ol	1,01
20.	16.691	Not identified	0,89
21.	16.816	Squalene	1,67
22.	17.102	Eicosane	0,62
23.	18.499	Vitamin E	0,66
24.	19.278	Campesterol	1,98
25.	19.522	Stigmasterol	4,06
26.	20.015	Stigmasterol	11,63
27.	20.390	alpha.-Amyrin	3,18
28.	20.705	9,19-Cycloergost-24(28)-en-3-ol, 4,14,-dimethyl-, (3.beta.,4.alpha.,5.alpha.) -	1,92
29.	20.824	alpha.-Amyrin	2,56
30.	21.317	Not identified	1,38
31.	22.197	Neophytadiene	1,71

The usage of gas chromatography/mass spectrometry method revealed in *Galega officinalis* extract chloroform fraction 31 compounds, from which fatty acids and their esters (39.10% of all compounds), diterpenes (6.16%), triterpenes (7.41%), phytosterols (19.59%) and flavonoids (2.89%) were identified.

**Fig. 2:**
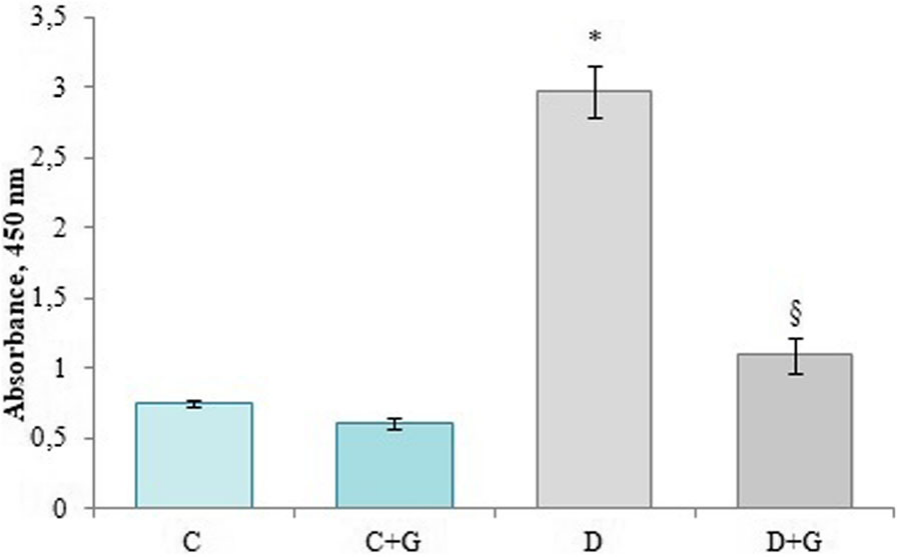
Effect of *Galega officinalis* extract on the proliferative activity of bone marrow cells of healthy and diabetic rats. * – *P* < 0.05 compared with controls. # – *P* < 0.05 compared with diabetic rats. § – *P* < 0.05 compared with control and diabetic rats

The level of thymidine analogue 5-bromo-2′-deoxyuridine inclusion in proliferating bone marrow cells was investigated using enzyme immunoassay method. A 3.9 times increase in proliferative activity of leukocytes in the S-phase of mitotic cycle take place under the DM conditions (Fig. 2).

It has been shown that administration of *Galega officinalis* extract to animals with DM caused the decrease by 2.8 times of 5-bromo-2′-deoxyuridine inclusion level (as compared to diabetes) in proliferating cells (Fig. 2). At the same time the extract did not produce a significant change in animal behavior or mortality.

The development of DM was accompanied by 44% increase of leukocytes precursors population in bone marrow, but the number of lymphocytes in the bone marrow does not undergo significant changes. Also, a 31% reduction in the number of myeloblasts which was the cause of the next decrease of juvenile and staff neutrophils number by 31% was shown (Table 2).

**Table 2:** The ratio (in %) of neutrophils and lymphocytes precursors in the bone marrow of control animals, animals with diabetes mellitus and under the condition of *Galega officinalis* extract administration

Cell type	C	C + G	D	D + G
*Myeloid lineage*				
Myeloblasts	1.91 ± 0.15	0.84 ± 0.09^*^	1.46 ± 0.12	1.26 ± 0.09
Metamyelocyte	18.54 ± 1.21	13.53 ± 0.89^*^	12.78 ± 1.39^*^	12.42 ± 1.17^*^
Juvenile (band) and staff (stab) neutrophils	15.67 ± 1.38	13.89 ± 0.28	10.84 ± 1.86	13.04 ± 0.83
Segmented neutrophils	1.58 ± 0.14	0.56 ± 0.07^*^	1.1 ± 0.15	0.67 ± 0.09^§^
Basophils of all types	0.19 ± 0.12	0.11 ± 0.08	0.44 ± 0.05	–
Eosinophils of all types	0.40 ± 0.06	0.37 ± 0.19	0.32 ± 0.08	0.41 ± 0.13
*Lymphoid lineage*				
Lymphoblasts	34.31 ± 1.74	38.56 ± 2.07	49.48 ± 2.67^*^	40.26 ± 1.94^§^
Lymphocytes	27.40 ± 3.03	32.13 ± 2.85	23.60 ± 2.36	30.95 ± 1.81^#^

^*^ – *P* < 0.05 compared with controls. ^#^ – *P* < 0.05 compared with diabetic rats. ^§^ – *P* < 0.05 compared with control and diabetic rats

In animals with DM *Galega officinalis* extract administration caused a decline in lymphoblasts number by 18.6% and an increase in lymphocytes amount by 31.1%. Moreover there was recorded the decrease in the number of juvenile and staff neutrophils (by 11.4%) and segmented neutrophils (by 64.6%) in the control animals treated with *Galega officinalis* extract compared to the control. Under the condition of extract administration to animals with DM a 39.1% decrease was found only in the number of segmented granulocytes, but a tendency to increase of juvenile and staff granulocytes content by 20.3%, versus DM was shown (Table 2).

**Fig. 3:**
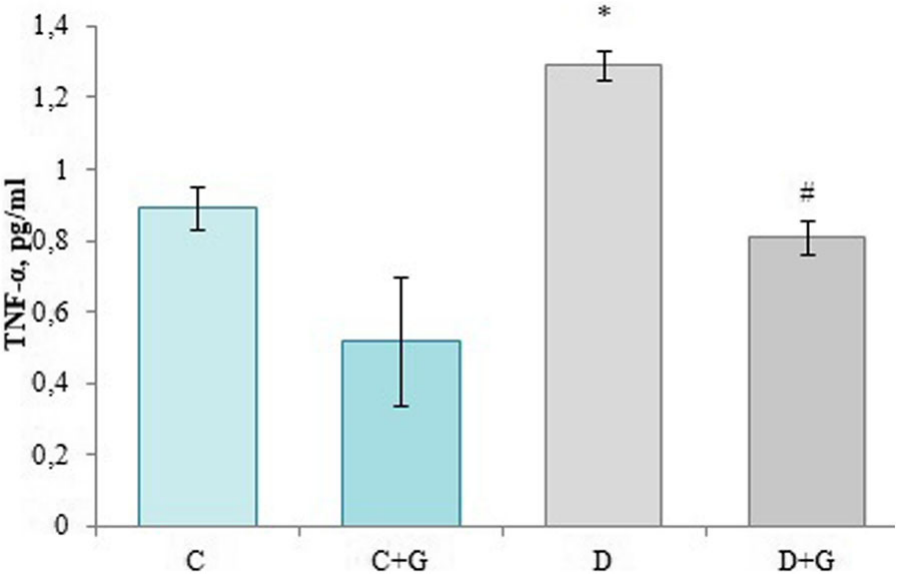
Effect of *Galega officinalis* extract on the level of TNF-α in plasma of healthy and diabetic rats

In group of animals with streptozotocin-induced DM was established the increase of TNF-α content by 44.9%, as compared to control values. Administration of extract to animals with DM caused 37.2% decrease of this cytokine concentration (Fig. 3).

**Fig. 4:**
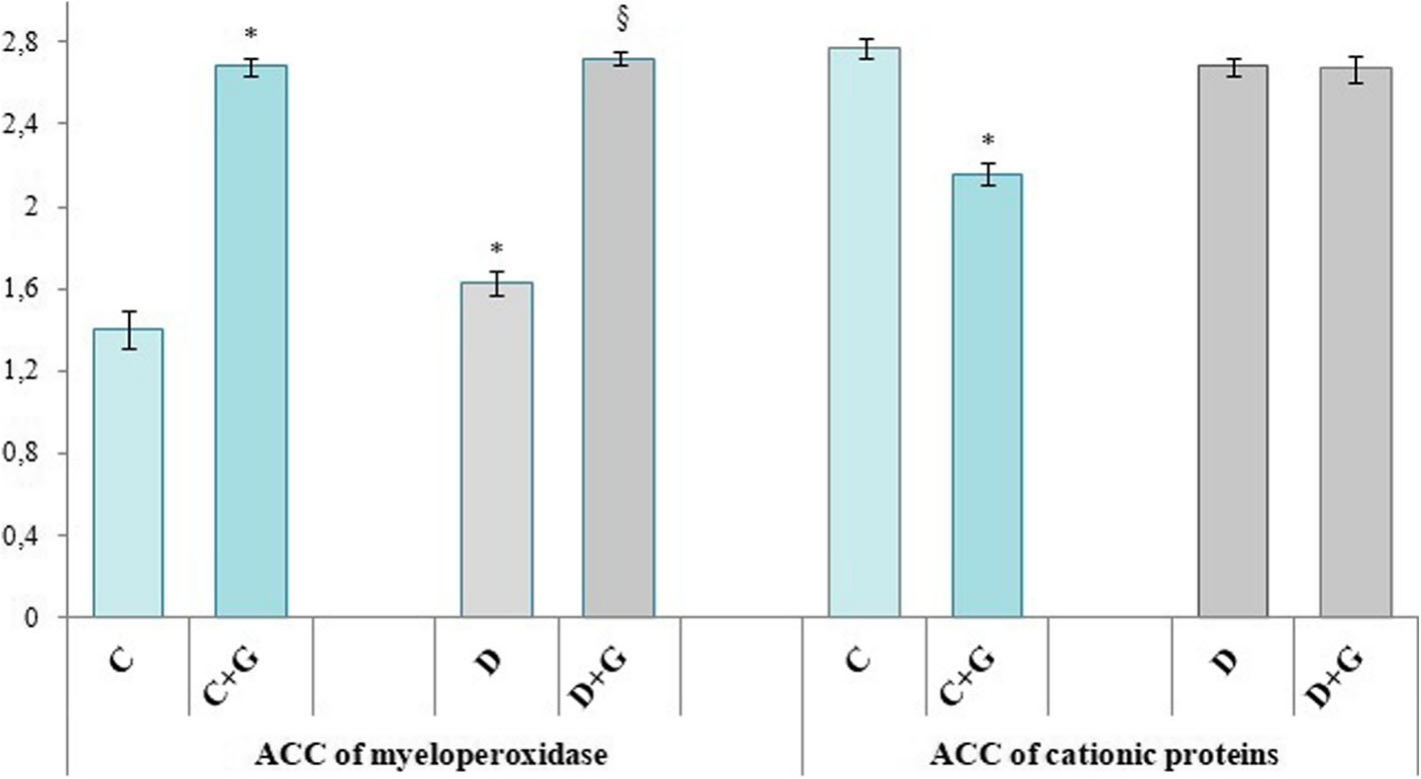
Changes in average cytochemical coefficient (ACC) of myeloperoxidase and cationic proteins under *Galega officinalis* extract administration in normal conditions and in the case of experimental diabetes mellitus

The development of DM was accompanied by the increase of the number of cells containing high amount of MPO, which in turn was accompanied by a growth rate of ACC by 16.4%, compared to control animals. *Galega officinalis* extract caused the reduction of ACC of myeloperoxidase by12.9% in animals with DM (Fig. 4).

The DM development is not accompanied by violation of nonoxygen-dependent microbe killing mechanisms mediated by cationic proteins, which is indicated by no significant change in ACC of cationic proteins. Administrations of *Galega officinalis* extract to animals with DM caused the reduction of ACC of cationic proteins by 34.3%. The extract had the similar effect when administrated to healthy animals, it was particularly demonstrated by 42.6% reduction of cationic proteins ACC, compared to control animals (Fig. 4).

**Fig. 5:**
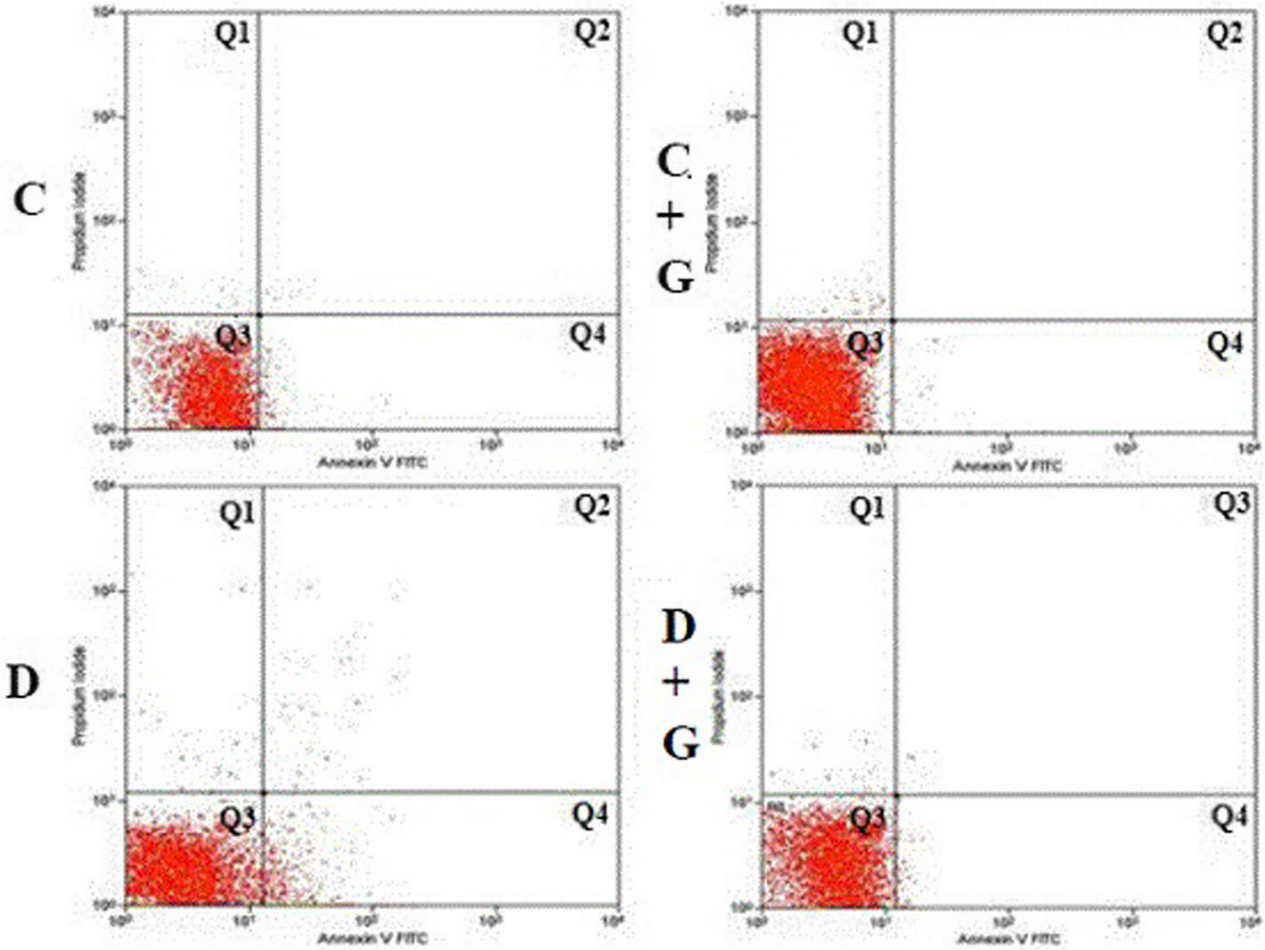
The two-dimensional histogram of apoptotic and viable lymphocytes distribution in DotPlot mode. On the horizontal axis - the intensity of annexin V - FITC fluorescence, the vertical axis - the intensity of PI fluorescence. Q3 (annexin V^−^/ PI^−^) - viable cells; Q4 (annexin V^+^/PI^−^) - cells with features of early apoptosis; Q2 (annexin V^+^/PI^+^) - cells with features of late apoptosis; Q1 (annexin V^−^/PI^+^) - cells with features of necrosis

The usage of dual fluorescent staining cells with annexin V, labeled with FITC and PI, makes it possible to carry out a detailed assessment of the severity of changes in apoptotic lymphocytes and to obtain quantitative distribution of blood cells in living cells, cells in the early stages of apoptosis and cell with apoptosis at late stages (Table 3, Fig. 5).

**Table 3:** Apoptotic lymphocytes detection by binding the surface of cells with annexin V and staining with PI under the administration of *Galega officinalis* extract in normal conditions and under experimental diabetes mellitus

Cell viability, %				
	C	C + G	D	D + G
Viable cells (annexin V^−^/ PI^−^)	96,41 ± 1,70	99,51 ± 0,07	87,56 ± 1,12^*^	98,12 ± 0,26^#^
Cells with features of early apoptosis (annexin V^+^/PI^−^)	2,75 ± 1,92	0,11 ± 0,04^*^	10,38 ± 0,58^*^	1,39 ± 0,31^#^
Cells with features of late apoptosis (annexin V^+^/PI^+^)	0,06 ± 0,02	0,004 ± 0,002	0,16 ± 0,08^*^	0,04 ± 0,02^#^
Cells with features of necrosis (annexin V^−^/PI^+^)	0,78 ± 0,30	0,38 ± 0,06^*^	1,91 ± 1,76	0,44 ± 0,07^#^

^*^ – *P* < 0.05 compared with controls. ^#^ – *P* < 0.05 compared with diabetic rats

Distribution histogram analysis based on viability showed that in control studied cell population was characterized by the predominant majority of living cells and the small number of apoptotic cells (96.41 and 2.81%, respectively). The development of DM was accompanied by almost four times (compared to the control) growing number of annexin-positive cells, indicating enhanced externalization of PS on the lymphocytes surface. At the same time the number of annexin-positive cells that also gave a positive reaction to propidium iodide staining increased by 2.8 times (Table 3, Fig. 5).

*Galega officinalis* extract administration leads to a reduction of lymphocytes with features of early apoptosis and late apoptosis (by 96% and 93.3%, respectively) in control group of rats and in animals with DM (by 86.6% and 75%, respectively) confirming its depressing effect on the genetically programmed cells death intensity of which is much enhanced in the studied pathology.

## Discussion

There is a number of evidence that show an association between inflammatory biomarkers and DM and the occurrence of its complications [18, 19]. The inflammatory process is accompanied by a growing number of effectors’ cells, among which white blood cells play the key role. Indeed it was proved that under the DM conditions an increase in proliferative activity of leukocytes takes place. In our opinion, it was interesting to examine the proliferation of which bone marrow cells populations were enhanced under the conditions of diabetes. With this purpose the ratio (in percentage) of neutrophils and lymphocytes precursors in the bone marrow was analyzed. The development of DM was accompanied by changes in leukocytes precursors population in bone marrow and reduction of the number of myeloblasts, which were the cause of the next decrease of juvenile and staff neutrophils number. This may be caused by early release of these cells in blood or the reduction of its formation. This can be mediated either through the changes in ratio of mediators, that stimulate the differentiation of precursor cells into specialized mature granulocytes, or through the modulation of the myeloid cells phenotype (especially by a decrease exposure of receptors to these mediators). Conflicting data regarding the blood cytokine levels exist in patients with DM. Plasma levels of proinflammatory and type 1 T-helper (Th1) cells cytokines, such as interleukin (IL)-1β, IL-2, IL-6, IL-12, tumor necrosis factor (TNF)-α, interferon (IFN)-γ, and granulocyte-macrophage colony-stimulating factor (GM-CSF) may be upregulated in patients with DM. However, other studies reported no difference in or even reduced production of these cytokines at the onset of DM. Nevertheless, the latest data using animal models shown that GM-CSF level was reduced in conditions of diabetes and its administration is capable to suppress many autoimmune diseases [20–22].

Inflammatory cytokines such as TNF-α are thought to play an important role in the pathogenesis of autoimmune type 1 diabetes. TNF-α is directly implicated in the destruction of β-cells in vitro and has profound inflammatory effects in vivo acting directly on antigen-presenting cells and autoreactive T lymphocytes [23]. Increase of TNF-α content under the condition of DM can interfere with the insulin signalling pathway, particularly by inhibiting the tyrosine phosphorylation of the insulin receptor and insulin receptor substrate-1 (IRS-1) in adipocytes. The lipolysis-stimulating effect of TNF-α leads to increased serum levels of free fatty acids, reducing insulin sensitivity, moreover TNF also has a direct inhibitory effect on insulin action in the liver. These TNF-α effects lead to reduced glucose uptake in muscle, and to increased hepatic glucose production. Recent data suggest that TNF-α also regulates expression of several adipocyte genes known to modulate insulin sensitivity/ resistance. TNF may even reduce β-cell function by direct effects, further contributing to its role in the development of diabetes [24].

Decrease of the neutrophil precursors may occure due to the growth of TNF-α content (Table 2, Fig. 3). TNF has inhibitory effects on granulocyte-macrophage precursors in vitro and on committed and primitive hematopoietic precursor in vivo. Specifically, an increased production of mature granulocytes and an increase in the number of committed granulocyte-macrophage progenitor cells (CFU-GM) in bone marrow of TNF-deficient mice was observed, suggesting a possible inhibitory role of TNF on myelopoiesis in vitro [25].

Under the condition of DM on the background of neutrophils precursor’s number reduction we have established an increase in the number of lymphocytes precursors – lymphoblasts by 44%. Simultaneously, the number of lymphocytes in the bone marrow does not undergo significant changes (Table 2). This may indicate an outing of lymphocytes immature forms in bloodstream under the condition of DM. Lymphocytes play a key role in the immune response that drives type 1 DM. It has been shown recently that single nucleotide polymorphisms is associated with DM and other autoimmune diseases and was enriched preferentially within cell super-enhancers. Intriguingly, superenhancer-associated genes show a striking enrichment for cytokines, cytokine receptors, and factors that regulate lymphocytes proliferation [26]. In addition, increased proliferation of lymphocytes in the conditions of DM may be caused by malfunction of signaling pathways involved in the lymphocyte proliferation such as protein kinase C and Toll-like receptor 4 [27].

On the other hand, TNF-α is a proinflammatory cytokine involved in cytokine cascade and leukocyte recruitment and a proimmune cytokine required for IL-12 and IFN-γ production, Th1 polarization and T cell differentiation [28]. Therefore the lymphocyte precursors number growth under the DM conditions may be due to the growth of the TNF-α content (Table 2, Fig. 3).

Considering the influence of biologically active substances of the *Galega officinalis* extract on the amount of different types of white blood cells and the apoptotic process, we find it suitable to analyze its effect on the immunocompetent cells proliferation.

Changes in leukocytes precursors’ content under the condition of *Galega officinalis* extract administration can be caused by the influence of biologically active substances of this extract on the production of TNF-α. The obtained result agree with other studies in which it has been shown that Fabaceae extract contains active compounds that reduce TNF-α and other inflammatory cytokines. It was demonstrated that Fabaceae lowers TNF-α levels by potently inhibiting the genetic transcription factor that activates TNF-α and IL-1B in the synovial tissue lining the joint [29].

The revealed influence of *Galega officinalis* extract on proliferative activity of leukocytes may be caused by the presence of inositol in its composition [17]. Inositol obtained from exogenous source is absorbed through the human digestive tract and internalized in the cells by active transport mechanisms. After the internalization, inositol phosphorylates to a form that can be easily transported via blood plasma to distant cells. In cells inositol is involved in the metabolism of phospholipids and is an important regulator of such vital cellular functions as cell proliferation, differentiation, and signal transduction [30].

It can be assumed that the established effect of investigated medicine on proliferative activity of leukocytes can be associated with the presence of fatty acids in the *Galega officinalis* extract [17]. When added exogenously fatty acids suppress the proliferation of leukocytes in whole blood in response to concanavalin A (Con A). Feeding rats diets rich in linolenic acid decreases spleen lymphocyte proliferation compared with feeding some other diets. In addition, it was established that an increased amount of linolenic acid in human diet resulted in a significant decrease in Con A-stimulated lymphocyte proliferation [31, 32]. Lymphocytes readily incorporate fatty acids into their lipids, and the presence of the excess of one fatty acid may result in accumulation of that particular fatty acid, leading consequently to modification of plasma membrane fatty acid composition and an alteration of membrane fluidity. Such changes could cause the observed decreases in proliferation [33]. Fatty acids, especially α-linolenic acid, contained in a significant number in the investigated extract [17], decreases IL-1 and TNF production by lymphocytes and monocytes [34, 35]. The mechanism for inhibition of TNF-α and IL-1 synthesis by dietary n-3 fatty acids is unknown, but may involve eicosanoid mediators because n-6 eicosanoid synthesis is inhibited by n-3 fatty acids, and n-6 eicosanoids can affect cytokine synthesis [36].

Another *Galega officinalis* extract components causing changes in proliferative activity of immunocompetent cells are flavonoids [17]. Anti-inflammatory effects of flavonoids may be linked to their ability to inhibit the lymphocytes proliferative response [37]. Flavonoids inhibit both cytosolic and membrane tyrosine kinase. Integral membrane proteins, such as tyrosine 3-monooxygenase kinase, are involved in a variety of functions, such as enzyme catalysis, transportation across membranes, and transduction of signals functioning as receptors of hormones and growth factors, and energy transfer in ATP synthesis. Inhibition of these proteins results in inhibition of cell growth and proliferation [38]. It was also shown that many flavonoids have inhibitory effect on TNF-α [39]. Recent studies have revealed that certain flavonoids, especially flavone derivatives, express their anti-inflammatory activity partly at least by modulation of proinflammatory gene expression such as cyclooxygenase-2, inducible nitric oxide synthase, and several pivotal cytokines. Flavonoids have different action mechanisms depending on their chemical structures. They probably have multiple cellular mechanisms acting on multiple sites of cellular machinery, but the most important contributors to anti-inflammation by flavonoids seem to be the effect on eicosanoid generating enzymes and the effect on the expression of proinflammatory molecules. The important moieties are the C-2,3-double bond, A-ring 5,7-hydroxyl groups, and B-ring 4′- or 3′,4′-hydroxyl groups. The C-3 hydroxyl group as in flavonols is favorable for LOX inhibition and oral anti-inflammatory activity. Flavones (without C-3- hydroxyl group) more strongly down-regulate proinflammatory gene expression [40].

Phytol is an important component of the extract that can influence the amount of TNF-α [17]. This compound exerted an anti-inflammatory action and inhibited polymorphonuclear cell migration, by decreasing TNF-α and IL-1β levels [41].

Among *Galega officinalis* extract components, squalene posses modulating effect on the content of TNF-α [17]. It was particularly shown that squalene reduced intracellular levels of reactive oxygen species (ROS), nitrites and such cytokines like TNF-α, IL-1β, IL-6 and IFN-γ, by abrogation of TNF-α, IL-1β, IL-6, IFN-γ, iNOS and COX-2 gene expression in LPS-activated human neutrophils and monocytes [42].

Also *Galega officinalis* extract contains a large amount of plant sterols among which are campesterol and stigmasterol [17]. It was investigated that these components also contribute to reducing TNF-α production [43].

Reduction of the TNF-α content under the conditions of studied extract administration to animals with DM can also be caused by α-amyrin [17]. Indeed it was shown that α,β-amyrin greatly prevented the production/release of the proinflammatory cytokines TNF-a, IL-1b and IL-6. This effect was clearly dependent on the activation of the cannabinoid system because the CB1R and CB2R antagonists significantly prevented the anti-inflammatory effects of α,β-amyrin [44].

Changes in immunocompetent cells proliferation necessarily entail a violation of the body immune defense, including the case of diabetes. Polymorphonuclear neutrophils (PMN) are connecting link between innate and adaptive immunity and performs a major role in antibacterial defenses. Investigators have established that phagocytosis and bactericidal activity of PMN are impaired under the condition of DM type 1 [1].

Among bactericidal agents present in neutrophil there are enzymes found in granules and metabolic products of the cell. After invagination of the neutrophil membrane and ingestion of the bacterium or immune complex, the azurophilic and specific granules fuse with the newly formed vacuole and discharge their contents. Microbe killing may be oxygen-dependent or independent. The nonoxygen-dependent mechanisms include the action of the enzymes contained in the granules, such as antibacterial cationic proteins, lysozyme, the various proteases, and the direct effects of lactoferrin. The oxygen-dependent mechanisms are again of two types: myeloperoxidase dependent and myeloperoxidase (MPO) independent. The oxidative response of the neutrophil results in transformation of molecular oxygen and hydrogen peroxide to free radicals, including the superoxide ion (О_2_^•–^), hydroxyl radical (OH^•^), and possibly singlet oxygen (O_2_^•^). These oxygen active metabolites are toxic for bacteria and fungi, and hydrogen peroxide, when combined with halide ions (Cl^−^, Br^−^) by myeloperoxidase, becomes an especially effective microbicidal agent [45].

We also investigated the amount of MPO in PMN as a part of oxygen-dependent mechanisms of microorganism killing,. MPO exerts potent and broad-spectrum microbicidal action against Gram-positive and Gram-negative bacteria, as well as yeast and fungi. Of the mammalian peroxidases, MPO is unique in its ability to catalyze the H_2_O_2_-dependent oxidation of Cl^−^ to OCl^−^. Such haloperoxidase activity is required for effective microbe killing. In addition to the requirement of H_2_O_2_ for OCl^−^ production, H_2_O_2_ also directly reacts with OCl^−^ to produce singlet molecular oxygen, a potent electrophilic oxygenating agent. The microbicidal action of MPO involves highly exergonic oxygenation reactions [46].

Increased MPO production by neutrophils from animals with DM can play an important role in vascular damage mediated by leukocytes. The excessive amount of MPO formed in neutrophilic granulocytes and excreted from them can interact with the vessel walls through various mechanisms – namely, binding and transcytosis across endothelial cells produce strong oxidant such as HOCl and HOBr, oxidation of nitric oxide and nitration of tyrosine. This could eventually mediate the development of the cardiovascular system diseases [47, 48].

In *Galega officinalis* extract inhibiting effect on MPO production may be predetermined by the presence and synergistic action of phytol [41], flavonoids [49], squalene [42], phytosterols [50] and amyrin [44].

We investigated the amount of antibacterial cationic proteins as part of nonoxygen-dependent microbe killing mechanisms,. Antimicrobial cationic proteins reach phagosomes from azurophil granules. Сationic proteins are able to form ion pores in membranes and can kill a variety of microorganisms, including bacteria, fungi and some viruses. Major antimicrobial cationic proteins that are present in PMN granules are defensins [51]. Defensins synthesis and release is regulated by microbial signals, developmental signals, and cytokines and in some cases by neuroendocrine signals in a tissue-specific manner. During phagocytosis, defensin-rich primary granules fuse with phagocytic vacuoles in which they generate high concentrations of defensins. Permeabilization of target membranes is the crucial step in defensinmediated antimicrobial activity and cytotoxicity. Conditions that interfered with permeabilization also prevented the loss of bacterial viability, indicating that permeabilization is essential for bacterial killing [52].

Administrations of *Galega officinalis* extract cause the reduction of ACC of cationic proteins, which can be associated with the presence of flavonoids in the composition of the extract [17]. It can be assumed that the inhibitory effect of *Galega officinalis* extract may be due to the presence of flavonoids. The exact mechanism of these substances influence on the cationic proteins content has not been established but it has been shown that that quercetin (at 10–50 μM) caused 70–90% inhibition of eosinophil cationic protein secretion [53].

Chronic hyperglycemia under the condition of diabetes mellitus leads to oxidative-nitrative stress during which the products exhibiting strong pro-apoptotic effect are formed. So the next stage of our work was to study the features of lymphocytes apoptosis of healthy rats and animals with DM on the background of *Galega officinalis* extract administration.

By means of flow cytometry method, the earliest events of apoptosis can be identified by recording the stage when the decision about transition of boundaries between viability and cell death are made [54]. The earliest event preceding apoptosis is oxidation of cell membrane lipid induced by the ROS elevated levels. This primarily refers to the polyunsaturated fatty acids in the composition of phospholipids represented in membranes mainly by phosphatidylserine. The formation of PS hydroperoxides violates its interaction with cytoskeleton proteins - annexins and facilitates translocation of oxidized PS from the inner on the outer plasma membrane. Therefore in the case of apoptosis induction PS appears on external side of the membrane [55, 56].

The expression of PS on the outer surface of the membrane occurs from early stages of apoptosis up to complete degradation of cell. It is used for differentiation of normal viable cells from those that are ready to apoptosis.

Changes in binding intensity of annexin V, labeled with FITC and PI, under the condition of diabetes, is a sign of membrane integrity violation and indicates the increase in the number of cells with early and late features of apoptosis.

Apoptosis of leukocytes can occur in two ways: the extrinsic or death receptor pathway and the intrinsic or mitochondrial pathway [57, 58].

Obviously, previously established by us increase of active forms of oxygen and nitrogen content in leukocytes [59], which has inherent damaging effect on subcellular structures, including the membranes of mitochondria and nuclei, accelerates apoptosis. The destruction of mitochondrial membranes under the influence of ROS can start the so-called intrinsic or mitochondrial pathway of apoptosis. The key event here is the release of cytochrome c from mitochondria intermembrane space, which joins to the adapter protein Apaf in the cytoplasm. This causes oligomerization of this protein and the formation of apoptosome. The latter activates initiational procaspases, which results in forming of proteolytic cascade that from a certain point makes process of apoptosis irreversible [60].

However, we do not exclude that the apoptosis we observed in our experiments was also activated by extrinsic or death receptor pathway. Under the condition of DM the content of proinflammatory cytokine TNF-α dramatically increases in serum (Fig. 3). It is known that TNF-α, reacting with transmembrane proteins, the so-called death receptors, attracts and activates procaspases that trigger the apoptotic cascade through the adapter proteins [61].

Corrective influence of *Galega officinalis* extract on the process of lymphocytes apoptosis in DM discovered by us correlated with changes in the content of TNF-α. Severity of lymphocytes apoptotic processes is consistent with the nature of the change of TNF-α content. It is possible that the protective action of the extract, to a great extent, can be realized through the inhibition of receptor-mediated apoptosis. The fact that *Galega officinalis* extract is capable of preventing the oxidative stress in lymphocytes can testify about the existence of additional mechanisms of its correcting effect. Particularly, another aspect of extract antiapoptotic effect realization may be an inhibition of mitochondrial apoptosis pathway - a process that is activated as a result of the occurrence of oxidative stress.

Established antiapoptotic effect of *Galega officinalis* extract is mediated by antidiabetic, antioxidant and anti-inflammatory properties of its components. In particular, the composition of the extract revealed a number of compounds that have potentially hypoglycemic (phytol, ethyl ester of palmitic acid, phytosterols - campesterol and stigmasterol, quinazolines derivatives), antioxidant (phytol, flavonoids, vitamin E) and anti-inflammatory (flavonoids, methyl ester of linolenic acid, α-amyrin) effect [17].

## Conclusions

Diabetes mellitus development was accompanied by violation of neutrophils and lymphocytes proliferation, increased activity of granulocytes MPO and enhanced lymphocytes apoptosis. Administration of *Galega officinalis* extract under the condition of DM promotes the restoration of neutrophils bone marrow pool, the reduction of lymphoblasts number and causes inhibition of the lymphocytes apoptosis process. Normalization of neutrophil functional competence by using a *Galega officinalis* can improve the course of the disease and in addition to their hypoglycemic action may prevent the development and progression of diabetes complications.

## Abbreviations

ACC: Average cytochemical coefficient
BrdU: 5-bromo-2′-deoxyuridine
CFU-GM: Granulocyte-macrophage progenitor cells
Con A: Concanavalin A
DM: Diabetes mellitus
FSC: Forward scattered light
GM-CSF: Granulocyte-macrophage colony-stimulating factor
IFN: Interferon
IL: Interleukin
IRS-1: Insulin receptor substrate-1
MPO: Myeloperoxidase
PI: Propidium iodide
PMN: Polymorphonuclear neutrophil
PS: Phosphatidylserine
ROS: Reactive oxygen species
SSC: Side scattered light
Th1: Type 1 T-helper cells
TNF: Tumor necrosis factor
